# Inulin Prebiotic Protects against Lethal *Pseudomonas aeruginosa* Acute Infection via γδ T Cell Activation

**DOI:** 10.3390/nu15133037

**Published:** 2023-07-05

**Authors:** Emilie Boucher, Caroline Plazy, Audrey Le Gouellec, Bertrand Toussaint, Dalil Hannani

**Affiliations:** 1 Univ. Grenoble Alpes, CNRS, UMR 5525, VetAgro Sup, Grenoble INP, TIMC, 38000 Grenoble, France; emilie.boucher@univ-grenoble-alpes.fr; 2 Univ. Grenoble Alpes, CNRS, UMR 5525, VetAgro Sup, Grenoble INP, CHU Grenoble Alpes, TIMC, 38000 Grenoble, France; cplazy@chu-grenoble.fr (C.P.); alegouellec@chu-grenoble.fr (A.L.G.); btoussaint@chu-grenoble.fr (B.T.)

**Keywords:** prebiotic, inulin, γδ T cells, *Pseudomonas aeruginosa*, immunity, gut–lung axis

## Abstract

*Pseudomonas aeruginosa* (*P. aeruginosa*) causes harmful lung infections, especially in immunocompromised patients. The immune system and Interleukin (IL)-17-producing γδ T cells (γδ T) are critical in controlling these infections in mice. The gut microbiota modulates host immunity in both cancer and infection contexts. Nutritional intervention is a powerful means of modulating both microbiota composition and functions, and subsequently the host’s immune status. We have recently shown that inulin prebiotic supplementation triggers systemic γδ T activation in a cancer context. We hypothesized that prophylactic supplementation with inulin might protect mice from lethal *P. aeruginosa* acute lung infection in a γδ T-dependent manner. C57Bl/6 mice were supplemented with inulin for 15 days before the lethal *P. aeruginosa* lung infection, administered intranasally. We demonstrate that prophylactic inulin supplementation triggers a higher proportion of γδ T in the blood, accompanied by a higher infiltration of IL-17-producing γδ T within the lungs, and protects 33% of infected mice from death. This observation relies on γδ T, as in vivo γδ TcR blocking using a monoclonal antibody completely abrogates inulin-mediated protection. Overall, our data indicate that inulin supplementation triggers systemic γδ T activation, and could help resolve lung *P. aeruginosa* infections. Moreover, our data suggest that nutritional intervention might be a powerful way to prevent/reduce infection-related mortality, by reinforcing the microbiota-dependent immune system.

## 1. Introduction

*Pseudomonas aeruginosa* (*P. aeruginosa*) is a common gram-negative environmental bacterium. This opportunistic bacterium causes nosocomial infections and is highly virulent in immunodeficient patients. Indeed, pulmonary infection with *P. aeruginosa* is one of the leading causes of death in ventilator-associated pneumonia (VAP), cystic fibrosis, and chronic obstructive pulmonary disease (COPD) patients [1,2,3]. Due to its adaptability, *P. aeruginosa* strains have become increasingly more resistant to antibiotics [4,5]. As a result, in 2017, the World Health Organization classified *P. aeruginosa* as an antibiotic-resistant “critical priority pathogen” among the bacteria species causing the greatest menace to the global population [6]. 

In an acute *P. aeruginosa* infection mouse model, several studies support the idea that the immune system, especially pulmonary γδ T lymphocytes, plays a key role in fighting such infection [7,8,9]. γδ T cells are unconventional innate T cells, primarily activated during *P. aeruginosa* infection [10]. This early activation makes them the first pulmonary producers of interleukin 17 (IL-17), and highly protective cells, during *P. aeruginosa* infection. In addition, lung γδ T cell infiltration is a good prognostic marker in the lethal *P. aeruginosa* acute infection mouse model and might, therefore, be an attractive therapeutic target [7,8,9,10]. Indeed, an active immune system is crucial for eliminating this opportunistic pathogen that has many virulence factors and easily acquires many antibiotic resistance determinants [5]. In immunodeficient patients, therapies and interventions reinforcing host immunity are therefore urgently required. 

The modulation of the immune system can be achieved in several ways, including the use of recombinant cytokines or monoclonal antibodies. Monoclonal antibodies can either act as agonists, activating receptors, or antagonists, inhibiting receptors or cytokines. These therapeutic strategies are currently being investigated in bacterial lung infections caused by *Mycobacterium tuberculosis* or *P. aeruginosa*, for example [11,12,13]. While vaccine approaches for *P. aeruginosa* infections have been ineffective so far, some immunotherapies, particularly monoclonal antibodies, are currently under investigation [14]. For over a decade, the gut microbiota has been shown to play a fundamental role in the induction, education, and function of the mammalian immune system [15]. The gut microbiota is composed of bacteria, fungi, archaea, and viruses that are in constant interaction with their host. Among these, bacteria have been the most extensively studied and have been shown to interact with host gut immunity [16,17]. Immune system modulation is balanced between pro- and anti-inflammatory signals produced by bacteria [17]. These signals include microorganisms-associated molecular patterns (MAMPs) and metabolites. Some metabolites derived from the gut microbiota can act as potent immune modulators [18]. These metabolites not only act on local (gut) immunity but also have an impact on distal, systemic immunity [15]. For instance, gut microbiota-derived aryl hydrocarbon receptor (AhR) ligands can influence the function of dendritic epidermal T cells, a type of T cell residing in the skin epidermis [19]. Additionally, short-chain fatty acids (SCFAs), which are metabolites produced through fiber fermentation by commensal bacteria, can directly modulate the function of various immune cells [20]. SCFAs can also enhance host hematopoiesis, promoting the generation of macrophage and dendritic cell (DC) precursors in the blood [21]. Consequently, they facilitate the migration of DCs with high phagocytic capacity to the lung [21]. These findings illustrate the gut–lung axis, where the gut microbiota and its derived metabolites potently regulate lung immunity under homeostatic conditions [22]. Since some studies have implicated the gut microbiome in certain bacterial pulmonary infection-associated diseases, targeting it could represent a promising therapeutic approach, particularly through nutritional interventions [23]. For example, COPD patients have an altered gut microbiome that correlates with disease features [24]. In the nontuberculous mycobacterial pulmonary disease (NTM-PDs) mouse model, modulation of the gut microbiome through L-arginine administration has shown a protective effect, even against multi-drug resistant *Mycobacterium abscessus* [25]. 

Based on these studies, the development of optimal strategies for modulating the composition and function of the gut microbiota for therapeutic purposes is highly appealing. As diet is one of the most potent modulators of the microbiota, nutritional intervention could significantly improve host anti-bacterial immunity [26]. A promising nutritional intervention approach involves diet supplementation with dietary fibers that exhibit prebiotic activity. Prebiotics are indigestible compounds found in vegetables, fermented by commensal bacteria of the gut microbiota [27]. A diet rich in prebiotics promotes the growth and metabolic activity of beneficial bacteria in the colon, thus favorizing gut-associated benefits [28,29]. Among prebiotics, inulin is a fructooligosaccharide (FOS) primarily found in chicory roots, composed of a fructosyl chain ending with a glucosyl moiety. Dietary supplementation with inulin has shown beneficial effect in several pathologies. In Type 2 diabetes patients, inulin consumption helps to regulate the glycemia [30]. In addition, dietary supplementation with inulin showed anti-tumor effect in several tumor-bearing mouse models [31]. Inulin is known to enhance the colonization of the gut by profitable bacteria, including *Bifidobacterium*, which has been described as an immuno-stimulatory species [32,33]. However, its biological mode of action still remains unknown. 

We previously demonstrated that a 15-day diet supplementation with inulin efficiently modulates the gut microbiota and promotes the growth of *Bifidobacterium* in mice [34]. This modulation of the microbiota led to the activation of colonic γδ T intraepithelial lymphocytes (IELs), notably enhancing their capacity to produce pro-inflammatory cytokines. Local γδ T cell activation was accompanied by systemic γδ T cell activation, as evidenced by enhanced cancer immunosurveillance. In contrast to mice on a control diet, mice supplemented with inulin exhibited a significant reduction in the size of subcutaneously transplanted melanoma tumors. This effect was dependent on the microbiota and γδ T cells, as simultaneous treatment with broad-spectrum antibiotics or administration of an anti-γδ TcR blocking antibody completely abolished the efficacy of inulin-mediated protection. Collectively, our findings demonstrate that this prebiotic strategy holds great potential for activating systemic host immunity, particularly γδ T cells, in a cancer context [34]. Considering the protective role of γδ T cells in *P. aeruginosa* infections [7,8,9], we hypothesized that prophylactic nutritional intervention with inulin supplementation could trigger a protective systemic activation of γδ T cell, potentially safeguarding against lethal *P. aeruginosa* infection. To evaluate this scenario, we conducted the present study.

## 2. Materials and Methods

### 2.1. Animals

Female C57Bl/6 mice (aged 5 weeks) were provided by Janvier SA Laboratory (Le Genest-Saint-Isle, France) and housed at “Plateforme de Haute Technologie Animale (PHTA)” UGA core facility (Grenoble, France), EU0197, Agreement C38-51610006, under specific pathogen-free conditions, in a temperature-controlled environment with a 12 h light/dark cycle and ad libitum access to water and diet. Animal housing and procedures were conducted in accordance with the recommendations from the Direction des Services Vétérinaires, Ministry of Agriculture of France, according to European Communities Council Directive 2010/63/EU, and according to recommendations for health monitoring from the Federation of European Laboratory Animal Science Associations. Protocols involving animals were reviewed by the local ethics committee, “Comité d’Ethique pour l’Expérimentation Animale no.#12, Cometh-Grenoble”, and approved by the Ministry of Research (APAFIS#21249-2019062715154462.v4, on 19 July 2019). Only female mice were used in this project, to avoid housing issues after group randomization. After a 1-week adaptation, the mice were randomly assigned to 2 groups, according to their diet.

(1) Healthy control group (Control), *n* = 16

*n* = 10: *P. aeruginosa* infected (*n* = 6 were used for survival assessment, and *n* = 4 were used for lung and blood-immunity analysis).

*n* = 6: Non-infected mice, used for lung and blood-immunity analysis.

(2) Inulin-supplemented group (Inulin) *n* = 24

*n* = 12: *P. aeruginosa* infected (*n* = 6 were used for survival assessment, and *n* = 6 were used for lung and blood-immunity analysis).

*n* = 6: *P. aeruginosa* infected + anti-γδ TcR, used for survival assessment.

*n* = 6: Non-infected mice, used for lung and blood-immunity analysis.

For inulin treatment, mice received a standard diet and drinking water supplemented with 7.2% inulin, starting 15 days before *P. aeruginosa* intranasal infection. Drinking bottles supplemented with inulin were renewed 3 times a week. All experiments were conducted with 6 mice per group, except when indicated in the figure legend.

### 2.2. Bacterial Culture

A clinical strain of *P. aeruginosa*, named CHA, was used for intrapulmonary infections [35]. Bacteria were cultured in Lysogeny Broth (LB) medium, under agitation at 37 °C. Bacterial growth, followed by optical density (OD) at 600 nm, was stopped at the exponential phase, corresponding to OD = 1. After centrifugation, bacteria were resuspended at a concentration of 1.25 × 10^8^ CFU/mL in Phosphate Buffer Saline 1X (PBS) (Gibco). The number of bacteria administered was confirmed by colony-forming unit (CFU) count after 24 h of culture at 37 °C on LB agar.

### 2.3. Intranasal Inoculation of P. aeruginosa

Mice were anesthetized with 4% isoflurane and infected intranasally with 3 × 10^6^ CFU *P. aeruginosa*, in 40 µL PBS. After 14 h, half of the mice were euthanized for pulmonary immunity analysis. The other half were under acute surveillance for 96 h. The severity of the symptoms was evaluated regularly by calculating a clinical score, as follows:

Score 1: normal clinical condition, may have slight piloerection, normal activity and no weight loss.
Score 2: slight piloerection, slight prostration, weight loss < 20%.
Score 3: piloerection, moderate prostration, weight loss < 20%, slightly closed eyes, irregular breathing, slightly reduced mobility, slightly reduced activity.
Score 4: piloerection, prostration, weight loss > 20%, closed eyes, reduced breathing rate, increased breathing depth, reduced activity: animal has reached moderate endpoint and should be euthanized.

When a score reached 3, animals were monitored every hour. Animals reaching a score of 4 were euthanized, to avoid any unnecessary suffering, and reported as dead from the infection.

### 2.4. In Vivo Blocking of γδ-TcR

Mice received 100 μg of the anti-γδTcR monoclonal antibody (clone UC7-13D5, Euromedex, Souffekweyersheim, France) intraperitoneally, in 100 µL PBS 1X. Anti-γδTcR antibodies were injected the day before and after the bacterial challenge. 

### 2.5. Lung Harvest

Lung lobes were collected in Roswell Park Memorial Institute (RPMI) complete medium (supplemented with 1% non-essential amino acids, 1 mM sodium pyruvate, 50 U/mL penicillin and 50 µg/mL streptomycin (all from Life technologies, Courtaboeuf, France)). Each lobe was lacerated into small pieces using scalpels, and digested with Liberase^TM^ (25 µg/mL, Roche, Meylan, France) for 30 min at 37 °C. Finally, the digested lungs were passed through a 70 µm cell strainer, washed, and cells were resuspended in 10% Fetal Bovine Serum (FBS) (Life technologies, Courtaboeuf, France) RPMI complete medium. 

### 2.6. Blood Sample Preparation

Blood was collected via retro-orbital sampling in K2E tubes (BD Medical, Le Pont-de-Claix, France). After centrifugation, blood pellets were resuspended in 1 mL Red Blood Cell Lysis buffer 1X (Sigma, Saint-Quentin Fallavier, France) and washed with 10% FBS complete RPMI.

### 2.7. Flow Cytometry

To allow intracellular cytokine detection, cell suspensions were stimulated for 4 h at 37 °C with 50 ng/mL phorbol 12-myristate 13-acetate (PMA) (Sigma, Saint-Quentin Fallavier, France), 1 µg/mL ionomycin (Sigma, Saint-Quentin Fallavier, France), in the presence of Golgi Stop^TM^ (BD Biosciences, Le Pont-de-Claix, France). Following activation, cells were stained for extracellular markers and incubated with 200 ng of each antibody for 15 min in the dark at RT. The antibodies targeting extracellular proteins were CD45 (30-F11), CD3 (17A2), CD4 (GK1.5) (Biolegend, Amsterdam, The Netherlands), γδ-TcR (eBioGL3 (GL-3, GL3)) (BD Biosciences, Le Pont-de-Claix, France). To allow intracellular labeling, cells were first permeabilized using a FoxP3 staining buffer kit (Life technologies, Courtaboeuf, France) before being incubated with intracellular antibodies for 1 h in the dark at RT. Antibodies targeting intracellular cytokines were IFNγ (XMG1.2) (Biolegend, Amsterdam, The Netherlands), IL-10 (JES5-16E3) (Life technologies, Courtaboeuf, France), and IL-17A (TC11-18H10) (BD Biosciences, Le Pont-de-Claix, France). After intracellular labeling, cells were fixed with FACS (fluorescence-activated cell sorting) Lysing Solution 1X (BD Biosciences, Le Pont-de-Claix, France) and stored at +4 °C until acquisition. All data were acquired on a BD Biosciences FACS Canto II and analyzed using FlowJo Software V10.4.2. The gating strategy is shown in Figure S1. 

### 2.8. Statistical Analysis

Statistical analyses were performed on GraphPad PRISM software. Mann–Whitney tests were used to compare 2 groups; ns corresponds to non-significant and * corresponds to *p*-value < 0.05.

## 3. Results

### 3.1. An Inulin-Enriched Diet Protects Mice against Lethal P. aeruginosa Infection and Enhances Pulmonary IL-17-Producing γδ T Cells

**Figure 1:**
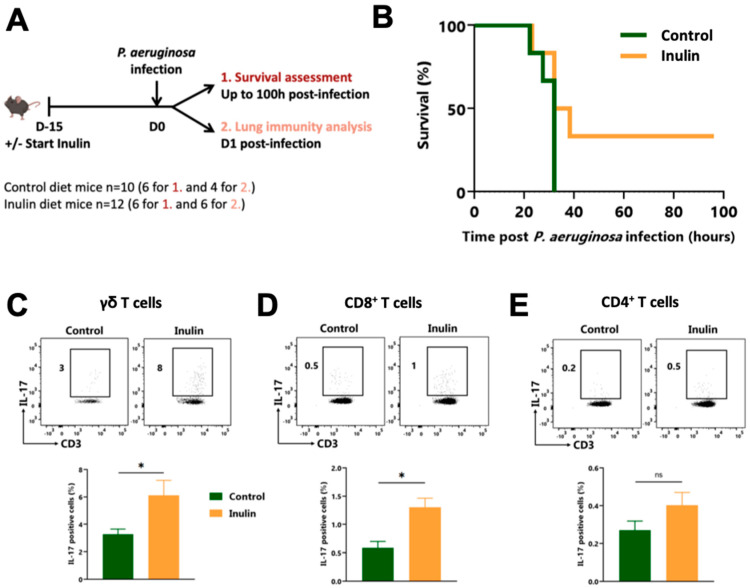
The inulin-enriched diet protects against lethal *P. aeruginosa* infection by reinforcing γδ T cells. (**A**) Experimental schedule. C57BL/6 mice were fed a control or an inulin-enriched diet (7.2% in drinking water) (*n* = 4–6 mice per group) starting 15 days before intranasal infection with 3 × 10^6^ CFU of *P. aeruginosa*. (**B**) Survival curves of mice treated and infected as described in (**A**). (**C**–**E**) Frequency of pulmonary IL-17-producing cells gated on CD45^+^ CD3^+^ γδ TcR ^+^, defined as γδ T cells (**C**), CD45^+^ CD3^+^ γδ TcR^−^ CD4^−^, defined as CD8^+^ T cells (**D**) or CD45^+^ CD3^+^ γδ TcR^−^, CD4^+^, defined as CD4^+^ T cells (**E**) from mice treated as in (**A**) 12 h post-infection. Graphs show the mean ± SEM. Statistically significant results are indicated by: ns = non-significant, * *p* < 0.05, by Mann–Whitney tests.

We have recently described how an inulin-enriched diet promotes systemic γδ T cell activation through microbiota modulation [19]. Since γδ T cells are critical players in controlling acute *P. aeruginosa* infections, we assessed the potential anti-bacterial effect of such an inulin-mediated immune boost. To do so, mice were fed either a standard diet or an inulin-enriched diet for 2 weeks before being intranasally challenged with a lethal dose of *P. aeruginosa* (Figure 1A). Only the two-week inulin-enriched diet efficiently protected 33% of infected mice from death (two out of six mice) (Figure 1B). 

To better understand the mechanisms behind this observation, we analyzed lung immunity by flow cytometry 12 h post-infection. While the inulin-enriched diet did not affect the infiltration of CD45^+^ and CD3^+^ in the lung (Figure S2), it significantly increased the proportion of pulmonary IL-17-producing γδ T lymphocytes (Figure 1C) and CD8^+^ T lymphocytes (Figure 1D), from 3% to 8%, and 0.5% to 1%, respectively. The frequency of IL-17-producing CD4^+^ T cells also tended to increase, although non significantly (Figure 1E). The proportions of Interferon γ (IFNγ)-producing T lymphocytes in the lungs were not affected by the inulin-enriched diet (Figure S3A), but the proportion of IL-10-producing CD8^+^ T cells was increased in the lungs of infected mice (Figure S3B).

**Figure 2:**
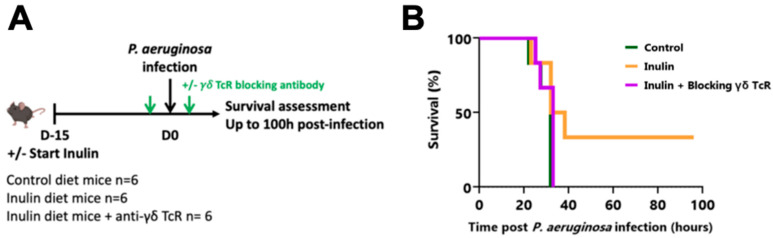
The inulin-mediated protective effect against *P. aeruginosa* depends on γδ T cells. (**A**) Experimental schedule. C57BL/6 mice were fed a control or an inulin-enriched diet (7.2% in drinking water) (*n* = 6 mice per group) starting 15 days before intranasal infection with 3 × 10^6^ CFU *P. aeruginosa*. Anti-γδ TcR antibodies were injected intraperitoneally (i.p.) into 6 mice from the inulin group the days before and after bacterial infection. (**B**) Survival curves of mice treated and infected as described in (**A**).

IL-17-producing γδ T lymphocytes have already been described as potent protective immune cells against *P. aeruginosa* infection. To confirm that the inulin-mediated protection was immune-dependent, and particularly dependent on γδ T cell-dependent, the same experiment was repeated, with one group receiving a blocking γδ TcR antibody the day before and the day after intranasal infection (Figure 2A). While 33% of inulin-treated mice were protected against *P. aeruginosa* infection (two out of six mice), the administration of the γδ TcR-blocking antibody completely abrogated this protective effect (Figure 2B), and none of the mice from either the control or the inulin + blocking γδ TcR group survived. These results collectively indicate that inulin triggered a Th17-polarized antibacterial lung immunity strongly supported by γδ T lymphocytes. Indeed, γδ T lymphocytes, and more likely their γδ TcR engagement, are mandatory for inulin’s protection against *P. aeruginosa*.

### 3.2. The Inulin Diet Reinforces Blood Circulating Immunity

**Figure 3:**
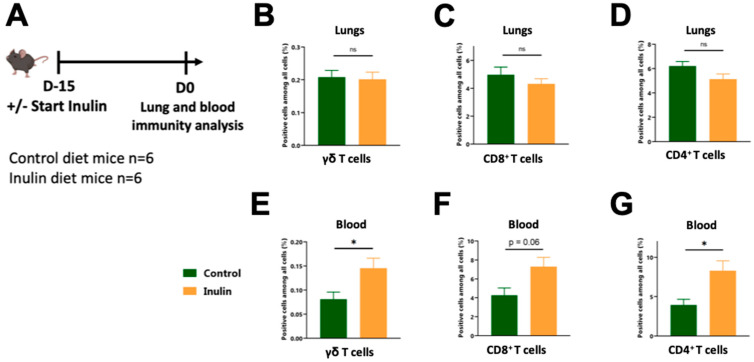
Pulmonary-activated T cells probably originate from the blood. (**A**) Experimental schedule. C57BL/6 mice were fed a control or an inulin-enriched diet (7.2% in drinking water) (*n* = 6 mice per group) starting 15 days before the analysis of the lung-infiltrated and blood-circulating immune cells. (**B**–**D**) Frequency of lung-infiltrated immune cells gated on CD45^+^ CD3^+^ γδ TcR^+^, defined as γδ T cells (**B**), CD45^+^ CD3^+^ γδ TcR^−^ CD4^−^, defined as CD8^+^ T cells (**C**) or CD45^+^ CD3^+^ γδ TcR^−^, CD4^+^, defined as CD4^+^ T cells (**D**) from mice treated as in (**A**). (**E**–**G**) Frequency of blood-circulating cells gated on CD45^+^ CD3^+^ γδ TcR^+^, defined as γδ T cells (**E**), CD45^+^ CD3^+^ γδ TcR^−^ CD4^−^, defined as CD8^+^ T cells (**F**) or CD45^+^ CD3^+^ γδ TcR^−^, CD4^+^, defined as CD4^+^ T cells (**G**) from mice treated as in (**A**). Graphs show the mean ± SEM. Statistically significant results are indicated by: ns = non-significant, * *p* < 0.05, by Mann–Whitney tests.

We have previously shown that a 15-day inulin-enriched diet triggers γδ T cell activation in the gut [34], specifically within colon IntraEpithelial Lymphocytes (IELs) γδ T cells. The gut microbiota can induce immune modulation in distal sites, including the lungs (known as gut-lung axis) [22]. Therfore, we investigated whether an inulin-enriched diet modulates lung γδ T cell infiltration under steady-state conditions or if these cells are only recruited from the periphery upon infection. To adress this, we analyzed both the immune cells infiltrating the lungs and the immune cells circulating in the blood following a 15-day inulin-enriched diet (Figure 3A), corresponding to the day of infection (Figure 1A). 

The inulin-enriched diet did not trigger higher lung leukocyte (CD45^+^) or T cell infiltration compared to mice on the control diet (Figure S4). Among the T cells, we did not observe any differences in the proportions of γδ, CD4^+^, and CD8^+^ T cells (Figure 3B–D). The cytokine patterns of lung-infiltrating T cells, particularly IFNγ, IL-17, and IL-10, were comparable in both groups (Figure S5A,B), except for the proportion of the IL-10-producing CD8^+^ T cells, which was found to be higher under the inulin diet (Figure S5C). While the inulin diet did not affect the proportions of pulmonary T lymphocytes, it significantly increased the proportion of γδ T and CD4^+^ T cells in the blood, from 0.08% to 0.14%, and from 3.9% to 8.3%, respectively (Figure 3E,G). The proportion of CD8^+^ T cells also showed a tendency to increase, but did not reach statistical significance (Figure 3F). Regarding the cytokine patterns of circulating T cells in the blood, the proportion of IL-17- and IL-10-producing T lymphocytes were comparable between both groups (Figure S5D,F), while the proportions of IFNγ-producing γδ and CD8^+^ T cells were reduced (Figure S5E).

Altogether, these observations suggest that the inulin diet promotes the circulation of γδ T cells and conventional T cells within the blood, but does not enhance their infiltration into the lung under steady-state conditions. However, upon acute infection, these cells are recruited to the lungs. 

## 4. Discussion

*P. aeruginosa* is widely recognized as a priority pathogen with respect to its high pathogenicity and the development of antibiotic resistance. There is an urgent need for emerging strategies that target the immune system, particularly those focusing on γδ T cells, which have been shown to play a critical role in combatting such infections [7,8,9]. In this study, we propose to harness the prebiotic and immune-stimulatory properties of inulin. It has been demonstrated, by ourselves and others, that this dietary fiber promotes the growth of immune-stimulatory bacteria such as *Bifidobacterium* [34,36], consequently leading to γδ T cell activation. Additionally, inulin promotes gut health by facilitating the production of SCFAs by the gut microbiota, which possess anti-inflammatory properties and support gut barrier integrity [37,38,39]. Strategies that reinforce the immune system through modulations of the gut microbiota are being increasingly investigated. Among the various gut microbiota modulators, inulin holds the advantage of being a widely consumed prebiotic, available either through diet source or as a supplement. 

In the present study, we report that an inulin-enriched diet has the capacity to protect 33% of mice against lethal infection with *P. aeruginosa*. This protective effect was accompanied by an increased proportion of IL-17-producing γδ T lymphocytes infiltrating the lungs, which play a critical role in this process, as blocking the γδ TcR in vivo abolished the protective effect mediated by inulin. Previous studies have also highlighted the importance of these cells in immune defense against *P. aeruginosa* infections [7,8,9], which our results confirm. Prior to infection, at a steady state, the proportion of immune cells in the lungs did not appear to be affected by inulin consumption. However, there was an increase in the proportion of patrolling T lymphocytes in the bloodstream. It is possible that the production of SCFAs or other metabolites induced by the inulin modulate γδ T cell proportion through the stimulation of lymphoid hematopoiesis, as has been described for the myeloid compartment [20,21]. As a result, an inulin-enriched diet enhances systemic immunosurveillance, making it more responsive to lung infiltration upon infection and thereby protecting the host from death. 

The molecular mechanisms underlying the activation of systemic γδ T lymphocyte are still unclear. In addition to establishing the essential role of γδ T lymphocytes as mediators, our data indicate that their activation is TcR-mediated rather than mediated through NKR (natural killer receptor) or TLR molecules. Since γδ T cells detect metabolites such as (E)-4-Hydroxy-3-methyl-but-2-enyl pyrophosphate (HMB-PP) derived from bacteria or isopentenyl-pyrophosphate (IPP) derived from tumors via their TcR [40], our findings strongly suggest that their activation may be mediated by microbiota-derived metabolites. Further research is needed to unravel the exact mechanisms and identify the specific metabolites that activate γδ T cells. The identification of these metabolites would be highly valuable for developing future post-biotic therapeutic strategies based on metabolites, particularly for immunocompromised or dysbiotic patients who are already infected, as nutritional interventions would require significant time to achieve the optimal modulation of the microbiota.

In addition to immune-stimulatory metabolites, we also hypothesized that anti-inflammatory and tissue repair signals (metabolites/mediators) could play a role. These signals would have a protective effect by preventing a harmful immune response from the host, which could lead to the destruction of lung tissue and, ultimately, death. Among the metabolites derived from inulin that have been described, SCFAs are known to promote the integrity of the gut epithelial barrier through IL-22 production, and possess anti-inflammatory properties [41]. In the protective effect observed in the acute *P. aeruginosa* infection mouse model due to inulin consumption, SCFAs could also regulate immunity, preventing mice from succumbing to excessive inflammation. Consistent with this, we observed a slight increase in the proportion of IL-10-producing CD8^+^ T cells in the lungs when the mice were on an inulin diet, supporting this hypothesis. We previously demonstrated that an inulin diet promotes IL-22, a cytokine involved in tissue repair, in the lamina propria of the gut [34]. It is conceivable that such modulation occurs at distant sites such as the lungs.

Beyond *P. aeruginosa* infection-related issues, the immune state of patients prior to any infection is a determining factor in clinical outcome. This has been exemplified by the severe acute respiratory syndrome coronavirus 2 (SARS-CoV-2) pandemic, where individuals with microbiota-related disorders (such as obesity and diabetes) were more prone to developing severe disease [42]. This can be attributed to pre-existing immune dysregulation (chronic systemic inflammatory state) and a compromised gut barrier, which facilitates the spread of the virus and subsequent multi-organ failure [43]. Both of these factors are associated with a dysbiotic state of the gut microbiota [44]. In a state of eubiosis, beneficial bacteria that are part of the microbiota produce key metabolites, notably SCFAs, which are crucial for gut health. SCFAs promote mucus production by goblet cells, preventing excessive inflammation [45]. The mucus acts as a barrier, shielding epithelial cells from bacteria and thus preventing tissue inflammation through Toll-like receptors (TLRs) and/or tissue damage caused by bacterial toxins [46]. SCFAs also possess anti-inflammatory properties [41], which help prevent autoimmunity and subsequent tissue damage. Additionally, SCFAs promote the expression of the tight junction protein by epithelial cells, maintaining the integrity of the intestinal barrier and preventing the translocation of bacteria or bacterial products from the gut lumen into the bloodstream [45]. On the other hand, dysbiosis, characterized by a loss or reduction of beneficial bacteria and decreased or absent SFCA production, leads to a thinner mucus layer, a decrease in anti-inflammatory signals, and compromised barrier integrity, due to the downregulation of tight junctions. Dysbiosis promotes local and systemic inflammation, particularly through bacterial product translocation. This systemic inflammation can trigger metabolic syndromes such as insulin resistance, diabetes, and obesity. It has been demonstrated that the initial response to SARS-CoV-2 infection is crucial in rapidly reducing viral load and preventing a later exacerbated immune response that could be detrimental and even fatal for patients [43]. However, individuals with metabolic syndromes may already have a pre-existing chronic inflammation that impairs the initial anti-SARS-CoV-2 response. Importantly, since the virus has a tropism for the gut due to the expression of the angiotensin-converting enzyme 2 (ACE2) in gut cells [47], a compromised gut epithelial barrier due to dysbiosis can facilitate viral propagation in the body. The virus can then reach vital organs, multiply, and cause death through immune exacerbation and multiple organ failure [43]. Similar scenarios could arise in future pandemics, especially if caused by an enterotropic pathogen (virus or bacteria) capable of eliciting exacerbated and harmful immune responses. Based on this knowledge and lessons learned, one can envision strategies to attenuate the severity and clinical outcome of viral and bacterial infections, as well as future pandemics, by promoting the optimization of the gut microbiota, particularly through nutritional intervention, within the general population. We believe that this should be given priority as a preventive strategy for various health issues, including infections. Inulin is a very interesting prebiotic for prevention, because it has been shown that this fiber could ameliorate mucus production, decrease gut permeability and reduce inflammation in obese patients [48]. Additionally, in line with our preclinical data, inulin profoundly modifies the composition of the gut microbiota in humans, in a bifidogenic manner [49]. Since inulin is a natural fiber, it is possible to enrich people’s daily diet in a simple way by increasing the intake of inulin-rich vegetables and fruits, and/or through supplementation. 

To conclude, our study has identified inulin as a promising fiber capable of triggering host immune reinforcement, which can protect certain individuals from lethal infections, in a microbiota- and γδ T cell-dependent manner. This research provides the groundwork for promoting a fiber-rich diet among the general population as a significant preventive strategy against current and future infection-related issues. In order to exploit these findings for severe acute infections, it is imperative that we urgently identify the microbiota-derived metabolites involved to develop post-biotic metabolite-based immunotherapy. 

## Supplementary Materials

The following supporting information can be downloaded at: https://www.mdpi.com/article/10.3390/nu15133037/s1, Figure S1: Flow cytometry gating strategy; Figure S2: CD45^+^ and CD3^+^ cell lung infiltration, upon *P. aeruginosa* infection. Figure S3: IFNγ- and IL-10-producing T cell lung infiltration, upon *P. aeruginosa* infection. Figure S4: CD45^+^ and CD3^+^ cell lung infiltration at steady state. Figure S5: Cytokine production by pulmonary and blood T Lymphocytes at steady state.
