# Supplementary material for: Inulin Prebiotic Protects against Lethal Pseudomonas aeruginosa Acute Infection via γδ T Cell Activation
Source: Nutrients. 2023 Jul 5;15(13):3037. doi: 10.3390/nu15133037 (PMC10346238; doi:10.3390/nu15133037)
Supplement: Supplementary file 1 [file nutrients-15-03037-s001.zip › nutrients-2466881-supplementary.pdf]

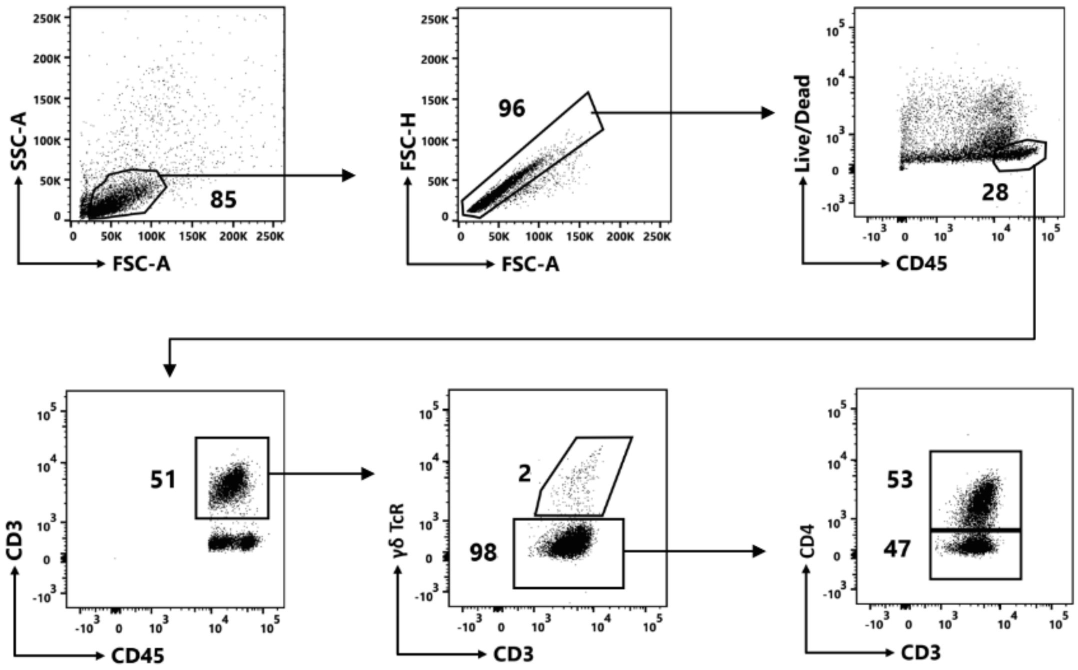

Figure S1 : Flow cytometry gating strategy
Illustrative dot plots showing the gating strategy allowing the analysis of lung cytokine-producing T lymphocytes. The same gating strategy was used for blood analysis.

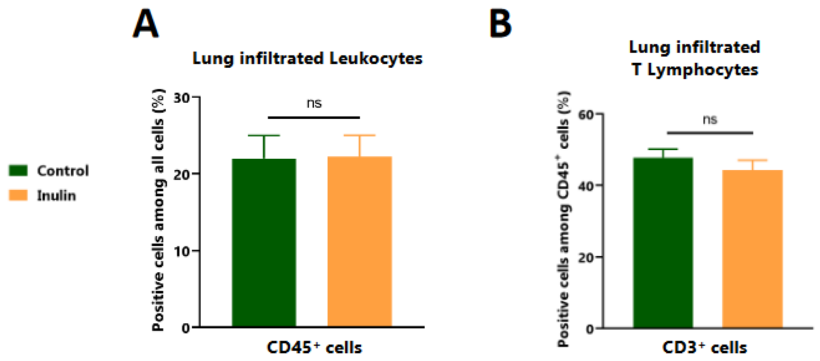

Figure S2 : $CD45^+$ and $CD3^+$ cell lung infiltration, upon *P. aeruginosa* infection. Frequency of (A) $CD45^+$ or (B) $CD3^+$ pulmonary cells from mice treated as in (Figure 1A). Graphs show the mean ± SEM. ns = non-significant by Mann-Whitney test.

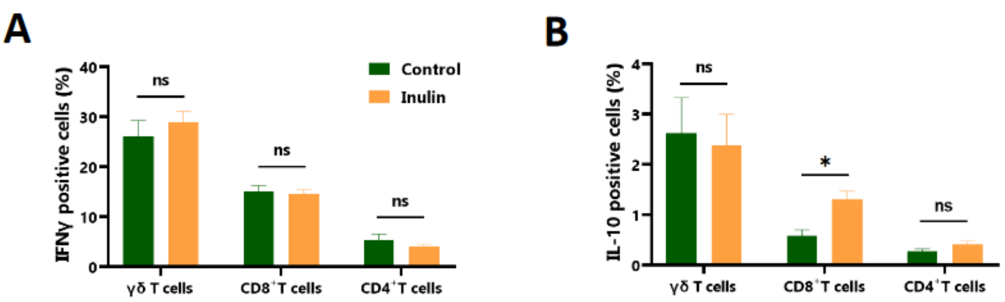

Figure S3 : IFNγ- and IL-10-producing T cell lung infiltration, upon *P. aeruginosa* infection. Frequency of pulmonary (A) IFNγ- or (B) IL-10-producing T cells from mice treated as in (Figure 1A) 12 hours post-infection. Graphs show the mean ± SEM. ns = non-significant, $*p < 0.05$ by Mann-Whitney tests.

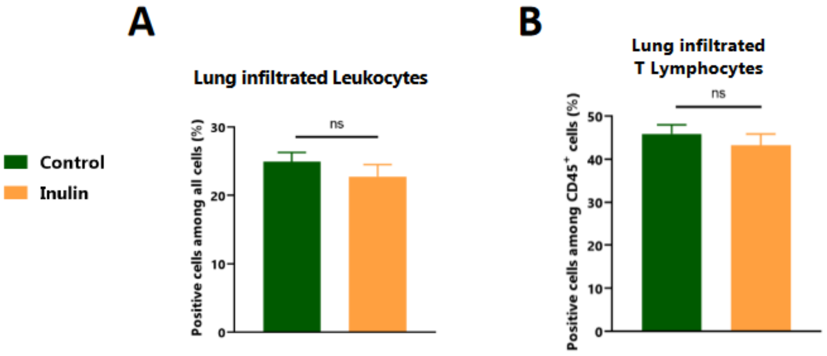

Figure S4 : CD45⁺ and CD3⁺ cell lung infiltration at steady state. Frequency of (A) CD45⁺ or (B) CD3⁺ pulmonary cells from mice treated as in (Figure 3A). Graphs show the mean ± SEM. ns = non-significant by Mann-Whitney test.

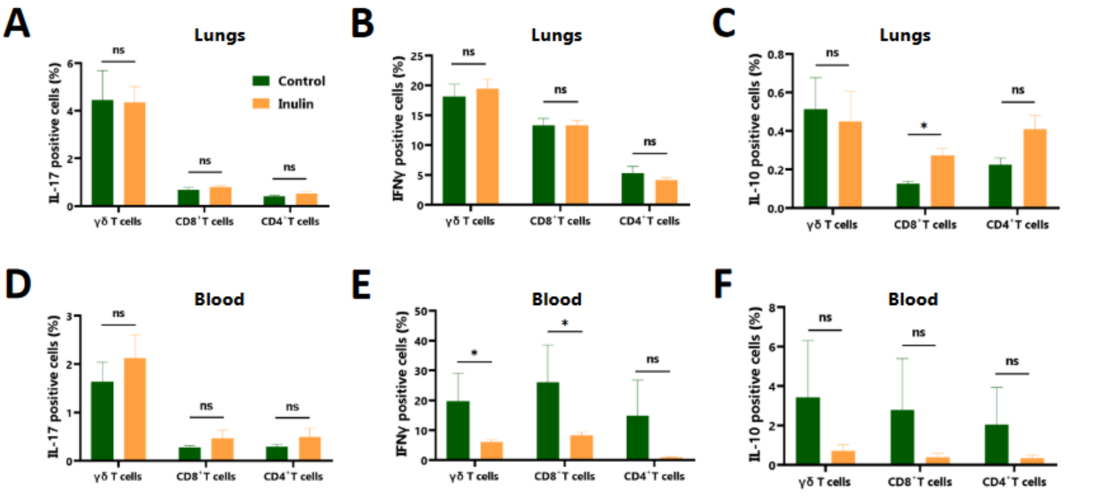

Figure S5 : Cytokine production by pulmonary and blood T Lymphocytes at steady state. (A-C) Frequency of pulmonary (A) IL-17, (B) IFNγ- or (C) IL-10-producing T cells from mice treated as in (Figure 3A). (D-F) Frequency of blood circulating (D) IL-17, (E) IFNγ- or (F) IL-10-producing T cells from mice treated as in (Figure 3A). Graphs show the mean ± SEM. ns = non-significant, *$p<0.05$ by Mann-Whitney test.
